# Priming exercise accelerates oxygen uptake kinetics during high-intensity cycle exercise in middle-aged individuals with type 2 diabetes

**DOI:** 10.3389/fphys.2022.1006993

**Published:** 2022-11-18

**Authors:** Joel Rocha, Norita Gildea, Donal O’Shea, Simon Green, Mikel Egaña

**Affiliations:** ^1^ Division of Sport and Exercise Sciences, Abertay University, Dundee, United Kingdom; ^2^ Department of Physiology, School of Medicine, Trinity College Dublin, The University of Dublin, Dublin, Ireland; ^3^ Endocrinology, St Columcille’s and St Vincent’s Hospitals, Dublin, Ireland; ^4^ School of Science and Health, Western Sydney University, Sydney, AU-NSW, Australia

**Keywords:** near-infrared spectroscopy, oxygen extraction, cycling, exercise tolerance, oxygen uptake slow component

## Abstract

**Background:** The primary phase time constant of pulmonary oxygen uptake kinetics ( $\overset{\cdot }{V}O_{2}$ *τ*_p_) during submaximal efforts is longer in middle-aged people with type 2 diabetes (T2D), partly due to limitations in oxygen supply to active muscles. This study examined if a high-intensity “priming” exercise (PE) would speed $\overset{\cdot }{V}O_{2}$ *τ*_p_ during a subsequent high-intensity cycling exercise in T2D due to enhanced oxygen delivery.

**Methods:** Eleven (4 women) middle-aged individuals with type 2 diabetes and 11 (4 women) non-diabetic controls completed four separate cycling bouts each starting at an ‘unloaded’ baseline of 10 W and transitioning to a high-intensity constant-load. Two of the four cycling bouts were preceded by priming exercise. The dynamics of pulmonary $\overset{\cdot }{V}O_{2}$ and muscle deoxygenation (i.e. deoxygenated haemoglobin and myoglobin concentration [HHb + Mb]), were calculated from breath-by-breath and near-infrared spectroscopy data at the vastus lateralis, respectively.

**Results:** At baseline $\overset{\cdot }{V}O_{2}$ *τ*_p,_ was slower (*p* < 0.001) in the type 2 diabetes group (48 ± 6 s) compared to the control group (34 ± 2 s) but priming exercise significantly reduced $\overset{\cdot }{V}O_{2}$ *τ*_p_ (*p* < 0.001) in type 2 diabetes (32 ± 6 s) so that post priming exercise it was not different compared with controls (34 ± 3 s). Priming exercise reduced the amplitude of the $\overset{\cdot }{V}O_{2}$ slow component (A_s_) in both groups (type 2 diabetes: 0.26 ± 0.11 to 0.16 ± 0.07 L/min; control: 0.33 ± 0.13 to 0.25 ± 0.14 L/min, *p* < 0.001), while [HHb + Mb] kinetics remained unchanged.

**Conclusion:** These results suggest that in middle-aged men and women with T2D, PE speeds $\overset{\cdot }{V}O_{2}$ *τ*_p_ likely by a better matching of O_2_ delivery to utilisation and reduces the $\overset{\cdot }{V}O_{2}$ A_s_ during a subsequent high-intensity exercise.

## 1 Introduction

Type 2 diabetes (T2D) is a major chronic condition with a concerning rapidly increasing global prevalence. Importantly, men and women with T2D demonstrate a consistent impairment in cardiorespiratory capacity reflected by a decreased peak oxygen uptake ( $\overset{\cdot }{V}O_{2}$ _peak_) (Green et al., 2015), that is an independent predictor of all-cause mortality (Wei et al., 2000). In addition, at the onset of moderate-intensity exercise a slowed primary phase time constant of pulmonary oxygen uptake ( $\overset{\cdot }{V}O_{2}$ ) kinetics ( $\overset{\cdot }{V}O_{2}$ *τ*_p_) is observed in young and middle-aged people with T2D (Bauer et al., 2007; Mac Ananey et al., 2011; O'Connor et al., 2012; Kiely et al., 2015; O'Connor et al., 2015). Similarly, recent evidence suggests that compared with controls, in middle-aged individuals with T2D $\overset{\cdot }{V}O_{2}$ *τ*_p_ is also slowed during exercise transitions from a moderate-intensity baseline to high-intensity (i.e work-to-work transitions) (Gildea et al., 2021b). While $\overset{\cdot }{V}O_{2}$ *τ*_p_ is a well-established key determinant of exercise tolerance (Jones and Poole, 2005; Goulding et al., 2021), the mechanisms for the constrained $\overset{\cdot }{V}O_{2}$ *τ*_p_ in T2D remain to be elucidated, but accumulating evidence suggests that impairments in oxygen supply to the active musculature (Padilla et al., 2006; MacAnaney et al., 2011; Kiely et al., 2014) and a subsequent mismatch of local O_2_ delivery to muscle $\overset{\cdot }{V}O_{2}$ (Bauer et al., 2007; Gildea et al., 2019; Rocha et al., 2019; Gildea et al., 2021b) play an important role.

The impediments in $\overset{\cdot }{V}O_{2}$ kinetics in T2D are also apparent during high-intensity exercise transitions initiated from rest or ‘unloaded’ baseline, with Brandenburg et al. (1999) showing a significantly slower mean response time (MRT) of the $\overset{\cdot }{V}O_{2}$ kinetics in females with T2D compared with BMI-matched controls. Nevertheless, Mac Ananey et al. (2011) showed a non-significant tendency for a slower $\overset{\cdot }{V}O_{2}$ MRT and $\overset{\cdot }{V}O_{2}$ *τ*_p_ (∼13% and ∼5% respectively) during high-intensity cycling transitions initiated from static rest in females with T2D of similar characteristics, compared with BMI-matched controls (Mac Ananey et al., 2011). Noteworthy, when transitions are initiated from static (instead of ‘unloaded’) rest, $\overset{\cdot }{V}O_{2}$ *τ*_p_ has been shown to be ∼15% longer when the time delay is not constrained (Whipp et al., 1982) as was the case therein (Mac Ananey et al., 2011), potentially influencing their findings.

Importantly, abrupt or sudden transitions to high-intensity activity (i.e. running, cycling or stair climbing) from rest or very light activity are akin to those in daily life (such as commuting to work), so, there is a need to examine interventions that may enhance the $\overset{\cdot }{V}O_{2}$ dynamic response during these exercise transitions in T2D. In this regard, studies in healthy active individuals presenting with an initial fast $\overset{\cdot }{V}O_{2}$ *τ*_p_ show that an acute prior bout of heavy-intensity “priming” exercise (PE) does not alter $\overset{\cdot }{V}O_{2}$ *τ*_p_ during subsequent high-intensity upright cycling exercise initiated from rest (Burnley et al., 2000; Burnley et al., 2001; Burnley et al., 2002a; Koppo and Bouckaert, 2002; Sahlin et al., 2005). This is likely because PE appears to facilitate muscle oxygen delivery rather than specific metabolic pathways and in these healthy active individuals $\overset{\cdot }{V}O_{2}$ *τ*_p_ seems limited by the later (i.e. intracellular energetics) (Gerbino et al., 1996; Sahlin et al., 2005; Jones et al., 2006). However, PE accelerates the MRT of the overall $\overset{\cdot }{V}O_{2}$ dynamic response in these healthy participants typically through an increase in the primary phase amplitude of the $\overset{\cdot }{V}O_{2}$ ( $\overset{\cdot }{V}O_{2}$ A_p_) and/or reducing the slow component amplitude of the $\overset{\cdot }{V}O_{2}$ , the latter being potentially related to the reduced requirement for type II muscle fiber activation after PE (DiMenna et al., 2008). On the contrary, when $\overset{\cdot }{V}O_{2}$ kinetics are further slowed as a direct consequence of impaired O_2_ delivery and reduced perfusion pressure to active muscles, as is observed during supine or prone high-intensity exercise (Rossiter et al., 2001; Jones et al., 2006; Goulding et al., 2017), PE accelerates $\overset{\cdot }{V}O_{2}$ *τ*_p_ in the respective subsequent bouts of high-intensity exercise, possibly due to improved blood flow distribution, and/or reduced muscle fatigue to active muscles (DiMenna et al., 2010).

Thus, considering that O_2_ supply to the muscle seems to be constrained in individuals with T2D, and high-intensity priming exercise has been proposed as an intervention that can augment the delivery of O_2_ to the muscle, we tested the hypothesis that PE would reduce $\overset{\cdot }{V}O_{2}$ *τ*_p_ in a subsequent bout of high-intensity exercise initiated from unloaded exercise in this population. Given that alterations exist in muscle fiber type in the T2D skeletal muscle, with individuals with T2D possessing larger proportions of type II and lower proportions of type I fibers than controls (Marin et al., 1994), we also hypothesized that in individuals with T2D PE would reduce the $\overset{\cdot }{V}O_{2}$ A_s_. To shed light on contributions of muscle fractional O_2_ extraction to any PE-induced changes in $\overset{\cdot }{V}O_{2}$ kinetics, this study measured rates of muscle deoxygenation (i.e., deoxygenated haemoglobin and myoglobin, [HHb + Mb]) using near-infrared spectroscopy (NIRS). In addition, the age of participants was limited to less than 60 years to control for the potential confounding effects of age on the T2D-induced effects on exercise tolerance (Wilkerson et al., 2011; O'Connor et al., 2015).

## 2 Methods

### 2.1 Participants and recruitment

A total of 22 individuals, 11 with T2D (7 men/4 women) and 11 healthy controls (7 men/4 women) volunteered and provided written informed consent to take part in this study (Table 1). Recruitment for the control group was undertaken from the general population, whilst individuals with T2D were recruited from outpatient diabetes clinics of two hospitals in Dublin (i.e. St. Vincent’s University Hospital and St. Columcille’s Hospital). Four of the women participating in this study were premenopausal (2 T2D and 2 Control) and four were postmenopausal (2 T2D and 2 Control) not undergoing hormone replacement therapy. All participants were non-smokers (not smoking during the previous 12 months) and physically inactive [(≤150 min week^−1^ of moderate-intensity (<ventilatory threshold, VT) exercise in the preceding 6 months] (McKay et al., 2022). The latter status was confirmed by participants wearing RT3 triaxial accelerometers (Stayhealthy Inc., CA) over a 5-day period (Table 1) (Rowlands et al., 2004). Participants’ time since diagnosis of T2D was between 2 and 10 years (mean ± SD = 4.5 ± 3.2 yrs) and had HbA_1c_ levels below 10%. In addition, exclusion criteria included the use of β-blockers or insulin, clinical evidence of liver or renal disease, if they suffered from persistent proteinuria (urine protein >200 mg/dl) or had high creatinine levels (suggestive of renal disease, which can alter exercise performance); autonomic insufficiency/dysfunction, symmetrical neuropathy, abnormal cardiac function, or evidence of ischaemic heart disease. Regarding medication, two individuals in the control group were on statins whilst participants with T2D were treated with either oral (n = 9) and/or subcutaneous (n = 2) hypoglycaemic agents (only metformin, *n* = 5; metformin and sulphonylurea, *n* = 1; SGLT-2 inhibitors, *n* = 1; GLP-1, *n* = 1), as well as antihypertensive medication (ACE inhibitor, *n* = 3; ARBs, *n* = 2; CCBs, *n* = 3) and statins (*n* = 6). The study received ethical approval from both Trinity College Dublin (TCD), and St Vincent’s Healthcare research ethics committees.

**TABLE 1:** Physical characteristics, peak exercise values, and activity levels.

	Controls	T2D	*p*-value
*n*	11	11	
Physical characteristics			
Sex (male, female), *n*	7, 4	7, 4	
Age, yr	40 (18)	43 (14)	0.21
Stature, m	1.70 ± 0.08	1.71 ± 0.09	0.77
BMI, kg/m^2^	30.9 ± 3.9	30.8 ± 4.6	0.97
Body Mass, kg	88 (17)	92 (33)	0.97
Fat layer VL, mm	7.9 (7.4)	6.0 (2.5)	0.25
HbA_1c_, %	5.1 ± 0.1*	7.4 ± 1.7	0.02
FPG, mmol/L	4.3 ± 0.7*	8.6 ± 3.5	0.01
Time since diagnosis, yr		4.5 ± 3.2	
$\overset{\cdot }{V}O_{2}$ , L/min	2.50 ± 0.52*	1.94 ± 0.49	0.02
$\overset{\cdot }{V}O_{2}$ , mL.kg^−1^.min^−1^	28.4 ± 6.6*	21.3 ± 3.59	0.01
PO_peak_, W	199 ± 53*	149 ± 43	0.03
Habitual physical activity			
Inactive, h/day	19.4 ± 1.6	18.4 ± 0.3	0.19
Light, h/day	3.7 ± 1.3	5.0 ± 0.3	0.06
MVPA, h/day	0.86 ± 0.60	0.69 ± 0.29	0.56

Data are means ± SD, for variables that were normally distributed and median with interquartile range in parentheses for variables that showed significant skewness and were not normally distributed in one or both groups. *n*, no. of participants. BMI, body mass index; VL, vastus lateralis; HbA_1c_, glycosylated haemoglobin; FPG, fasting plasma glucose; MVPA, moderate-to-vigorous physical activity; $\overset{\cdot }{V}O_{2}$ , oxygen uptake; PO, power output. *Significantly different from T2D (*p* < 0.05).

### 2.2 Experimental procedures

#### 2.2.1 Overview

Upon successful completion of a treadmill stress test (Bruce protocol) at St Columcille’s Hospital, participants attended the testing laboratories on two separate occasions. The participants in the control group completed all tests in the University’s human performance laboratory whilst participants with T2D did so in the exercise testing facility at St Columcille's Hospital. Visit one consisted of participants completing a maximal cycling test to exhaustion to measure peak oxygen uptake ( $\overset{\cdot }{V}O_{2}$ _peak_). In visit 2, participants completed four exercise transitions from a baseline of 10 W (i.e. unloaded) to high-intensity, of which two were preceded by PE. Cycling tests were completed in the upright position on an Excalibur Sport cycle ergometer (Lode B.V, Groningen, Netherlands). Participants were asked to avoid the consumption of caffeine, alcohol and non-prescribed nutritional supplements together with any arduous physical activities during the 24 h preceding each visit. Menstrual cycle was controlled for when scheduling the visits of the premenopausal participants in this study, with testing taking place during the mid-follicular phase of their menstrual cycle (days 5–12, self-determined).

#### 2.2.2 Visit 1: Maximal cycling test to exhaustion

All participants completed a ramp incremental cycling test to volitional exhaustion with an initial work rate of 10 W for 2 min (i.e., ‘unloaded’ cycling) followed by a progressive increase in power output of 10–25 W/min (based on each individual’s physical activity level). Participants were required to maintain a constant cadence throughout the test, self-selecting a pedalling rate between 60 and 75 rpm. Test termination occurred when participants had a cadence reduction of 10 rpm for more than 5s. At the end of the test peak work rate was determined as the highest power output achieved, whilst $\overset{\cdot }{V}O_{2}$ was determined as the highest $\overset{\cdot }{V}O_{2}$ value (15-s average) attained. The V-slope method was used to determine VT (Beaver et al., 1986).

#### 2.2.3 Visit 2: Four cycling exercise transitions

**FIGURE 1:**
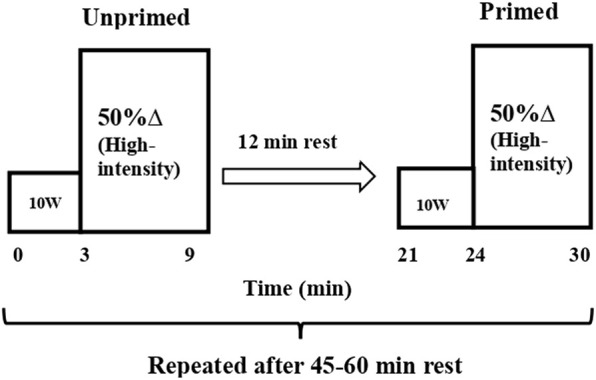
Schematic representation of the protocol. Unprimed and primed cycling step transitions performed at high-intensity cycling exercise (Δ50%; the sum of the power output at VT and 50% of the difference between the power output at VT and $\overset{\cdot }{V}O_{2}$ _peak_). All step transitions, each lasting 6 min, were preceded by 3 min of cycling at 10 W (i.e. ‘baseline’ cycling). Unprimed and primed transitions were separated by 12 min of passive rest. The 2 step transitions (unprimed and primed) were repeated following 45–60 min of passive rest within the same laboratory visit.

All participants completed four identical 9-min cycling exercise bouts transitioning from an ‘unloaded’ power output of 10 W (3 min) to a constant-load of 50% delta (50%Δ) (6 min). The latter intensity was determined from the results of the maximal cycling test by adding the 50% difference between the power outputs at $\overset{\cdot }{V}O_{2}$ and VT to the power output at VT. All participants completed the four bouts of exercise in the same order. Two of the bouts (bouts 1 and 3) were carried out without PE (50%Δ unprimed) and two bouts (bouts 2 and 4) were completed preceded by PE (50%Δ primed) (Figure 1). The unprimed 50%Δ bouts were used as PE. A resting period of 12 min was used between the first and second bouts, and the third and fourth bouts; whilst a 45–60 min seated rest period was used between the second and third bouts. The longer resting period was important to ensure that participant’s physiological parameters returned to a baseline state so as not to affect subsequent $\overset{\cdot }{V}O_{2}$ kinetics parameters. This was determined by measuring these parameters in a subgroup of 11 participants with T2D, albeit employing a single high-intensity transition which is consistent with previous reports in healthy active individuals (Burnley et al., 2006). Participants’ gas exchange/ventilatory variables, muscle oxygenation/deoxygenation and heart rate (HR) data were measured continuously throughout each exercise bout.

### 2.3 Measures

#### 2.3.1 Pulmonary gas exchange and heart rate

Breath-by-breath data was continuously obtained during exercise by participants wearing a facemask that was connected to a metabolic gas analysis system (Innocor, Innovision A/S, Odense, Denmark). Parameters analysed were oxygen consumption, carbon dioxide production, respiratory exchange ratio and minute ventilation. Calibration of equipment was undertaken before each use according to the recommendations of the manufacturer. In addition, calibration of the system’s oxygen and photoacoustic sensors is undertaken periodically (every 6–12 months) by the manufacturer. A heart rate monitor was used to measure HR at 5 s intervals (Polar S610i, Polar Ltd., Finland).

#### 2.3.2 Muscle deoxygenation and tissue oxygenation index

Muscle oxygenation (O_2_Hb + Mb), deoxygenation (HHb + Mb) and tissue oxygenation index (TOI) data were acquired using a continuous wave NIRS system (Niro 200NX; Hamamatsu, Japan). This device uses the spatially resolved spectroscopy (SRS) technique and modified Beer-Lambert (MBL) principle. Detailed information about this technique and its application during exercise is available elsewhere (Ferrari et al., 2011). In the present study this measurement was undertaken in the vastus lateralis (VL) muscle of the participant’s right quadriceps given the VL is the primary locomotor muscle during leg cycling (Laplaud et al., 2006; Okushima et al., 2016). In order to ensure good quality signals, necessary skin preparation was undertaken involving shaving any hair present and cleaning the area with alcohol. After the skin was dried, the probes in their rubber holder were securely positioned on the muscle, between 10 and 16 cm above the femoral condyle using transparent adhesive tape. A dark elastic bandage was also used to further protect the probes from external light and movement. The depth of the area being measured is approximately one-half the distance between the emitter and the receiver probes (∼1.5 cm). Therefore, ultrasound measurements of the skin and adipose tissue at the probe location were taken in all participants using the B-mode of a 2D ultrasound (Zonare Ultra Smart Cart, Software version 4.7, United States) to ensure that data collected was representative of the muscle tissue. This was confirmed with all participants having less than 1.5 cm of adipose tissue thickness at the probe location.

### 2.4 Data analysis

#### 2.4.1 Oxygen uptake kinetics

The linear interpolation method was used to estimate second by second values from the breath-by-breath $\overset{\cdot }{V}O_{2}$ data for each transition. Data was then aligned to ensure that the start of the exercise bout was time 0. To achieve a single smoothed averaged response for each participant, data were ensemble- and time-averaged into 5s bins. A biexponential (Eq. 1) function was then used to fit these responses:

$$\dot{V}O_{2}\left(t\right)=\dot{V}O_{2}baseline+A_{p}\left[1-e^{-(t-TDp)/\tau p)}\right]F1+A_{s}\left[1–e^{-(t\;–\;TDs)/\tau s)}\right]F2$$ (1)

In the above function $\overset{\cdot }{V}O_{2}$ (*t*) is $\overset{\cdot }{V}O_{2}$ (absolute) at a given time (*t*); $\overset{\cdot }{V}O_{2}$ baseline represents the mean $\overset{\cdot }{V}O_{2}$ in the last 30 s of the “unloaded” cycling; Amplitudes (A), time delays (TD) and time constants (*τ*) for $\overset{\cdot }{V}O_{2}$ primary (*p*) and slow component (s) phases are represented as A_p,_ A_s,_ TD_p_, TD_s_, *τ*_p_, and *τ*_s_ respectively. Time constant is the time that $\overset{\cdot }{V}O_{2}$ takes to reach 63% of the amplitude of the corresponding phase. F1 and F2 are two conditional expressions that ensure the fitting of the phase is restricted to the period at and beyond the time delay associated with that phase. The initial 20 s of the $\overset{\cdot }{V}O_{2}$ response data from the start of the cycling bout (i.e. cardiodynamic phase) were omitted but the TD_p_ was allowed to vary freely so that the fit could be optimised (Murias et al., 2011). A monoexponential curve was fitted to calculate mean response time (MRT) and ascertain the overall $\overset{\cdot }{V}O_{2}$ kinetics during high-intensity cycling irrespective of the different $\overset{\cdot }{V}O_{2}$ phases. A weighted nonlinear least-squares regression (TableCurve 2D, Systat, United States) was used to fit all $\overset{\cdot }{V}O_{2}$ response data. During the initial fit of the model, only data points within the 95% prediction interval were included. The average of the final 30 s of the $\overset{\cdot }{V}O_{2}$ was calculated to represent the end of exercise $\overset{\cdot }{V}O_{2}$ response (i.e. $\overset{\cdot }{V}O_{2}$ End A). The latter was then used to calculate the absolute A_s_ that is ( $\overset{\cdot }{V}O_{2}$ baseline + A_p_) subtracted from $\overset{\cdot }{V}O_{2}$ End A, while TD_s_ was constrained. The A_s_ was also calculated relative to the entire response (A_s_/(A_p_ + A_s_)). The end of exercise $\overset{\cdot }{V}O_{2}$ gain, representing the functional gain of the overall response, was also calculated by subtracting $\overset{\cdot }{V}O_{2}$ baseline from $\overset{\cdot }{V}O_{2}$ End A and normalised to the difference in power outputs between the unloaded and high-intensity cycling.

#### 2.4.2 [HHb + Mb] kinetics and tissue oxygenation index

The muscle deoxygenation (i.e. Δ[HHb + Mb]) response during high-intensity cycling was collected at a frequency of 1 Hz, was ensemble-averaged and time averaged into 5 s bins for each participant and was fitted using the same biexponential function (Eq. 1). TD was determined by visually inspecting a consistent rise from the pre-transition level since the Δ[HHb + Mb] is known to present a TD at the start of exercise before increasing in an exponential manner. This phenomenon has been suggested to reflect a close association between local oxygen delivery and muscular oxygen consumption (DeLorey et al., 2003; Grassi et al., 2003). Muscle deoxygenation data was therefore fitted from the TD onwards. The sum of TD and *t* was used to determine the effective response time (τ′Δ[HHb + Mb]) representing the time course for the primary phase of the Δ [HHb + Mb] response. Baseline TOI was calculated as the 30 s preceding each transition, and the End TOI as the final 30 s of exercise. ΔTOI was calculated by subtracting baseline TOI from End TOI.

#### 2.4.3 Heart rate kinetics

The heart rate data was fitted using a monoexponential function (Eq. 2) with the fitting window constrained to $\overset{\cdot }{V}O_{2}$ TD_s_:

$$HR\left(t\right)=HR\;baseline+A\left[1-e^{-(t-TD)/\tau )}\right]$$ (2)

In the above function HR baseline represents the mean HR in the last 30 s of the “unloaded” cycling.

### 2.5 Statistical analysis

The Shapiro-Wilk’s test was used to assess the normal distribution of the data. Between group comparisons of participants’ characteristics and peak performance data were undertaken using an unpaired Student’s t-test (for parametric data) or a Mann-Whitney U test (for non-parametric data). A two-way mixed model ANOVA [condition (unprimed, primed) x diabetes status (T2D, Control)] and the post hoc Tukey test were used to analyse all the dynamic response data for oxygen uptake, heart rate and muscle deoxygenation as well as TOI responses. *p* < 0.05 was used to determine statistical significance. Results from parametric analyses are presented as mean ± SD whereas non-parametric results are presented using median and interquartile ranges.

## 3 Results

### 3.1 Participant characteristics

Unsurprisingly, individuals with T2D had significantly higher HbA_1c_ and fasting plasma glucose levels than healthy individuals (Table 1). Importantly, the T2D and control groups were matched according to sex distribution, age, BMI, body mass and activity levels.

### 3.2 Peak exercise responses

Individuals with T2D had significantly lower absolute $\overset{\cdot }{V}O_{2}$ _peak_, $\overset{\cdot }{V}O_{2}$ _peak_ normalised to body mass, and peak power output than healthy individuals (Table 1).

### 3.3 Effect of priming exercise on oxygen uptake kinetics

**FIGURE 2:**
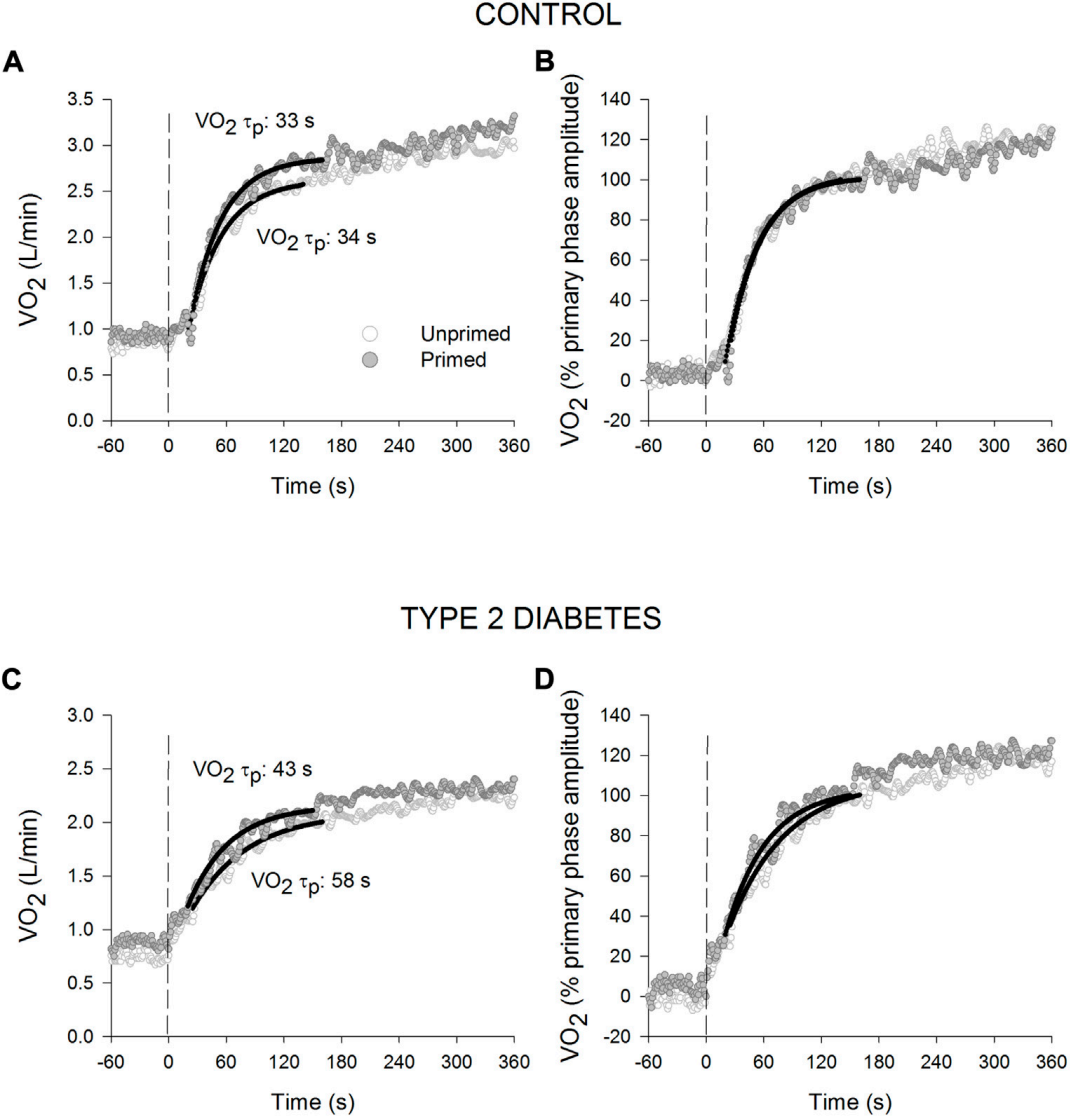
Oxygen uptake ( $\overset{\cdot }{V}O_{2}$ ) responses for a representative healthy control (**(A)**: absolute values; **(B)**: normalised to the % primary phase $\overset{\cdot }{V}O_{2}$ amplitude) and an individual with type 2 diabetes (**(C)**: absolute values; **(D)**: normalised to the % primary phase $\overset{\cdot }{V}O_{2}$ amplitude) during high-intensity cycling transitions without priming exercise (open circles) and with priming exercise (solid circles). The vertical line illustrates the abrupt transition to the higher work rate. The continuous lines of best fit illustrate the primary phase of the oxygen uptake ( $\overset{\cdot }{V}O_{2}$ ) response. Note the relatively slower response of the primary phase of the $\overset{\cdot }{V}O_{2}$ response in the unprimed compared with the primed bout in T2D.

**FIGURE 3:**
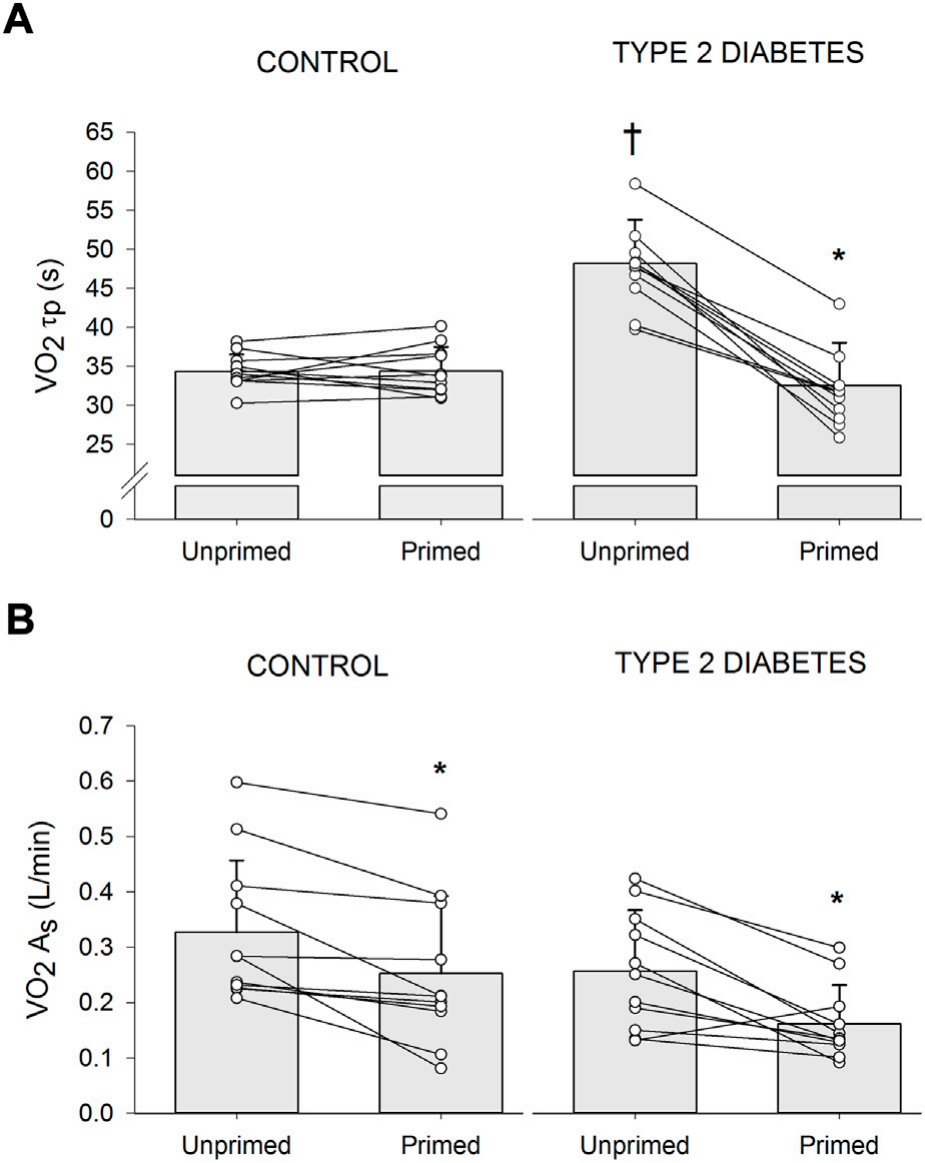
Individual and mean ± SD (*bar graph*) changes in time constant of the primary phase of oxygen uptake ( $\overset{\cdot }{V}O_{2}$ *τ*_p_) **(A)** and amplitude of the $\overset{\cdot }{V}O_{2}$ slow component ( $\overset{\cdot }{V}O_{2}$ A_s_) **(B)** in participants with type 2 diabetes (*n* = 11) and healthy controls (*n* = 11) during high-intensity cycling transitions without priming exercise (unprimed) and with priming exercise (primed). *p < 0.05 vs. unprimed within the same diabetes status group (i.e., within controls or within Type 2 diabetes). ^†^ *p* < 0.05 vs. healthy controls within the same condition (i.e., within unprimed or primed).

The primed and unprimed dynamic response characteristics of $\overset{\cdot }{V}O_{2}$ at high-intensity cycling exercise transitions for each group are presented in Table 2. The $\overset{\cdot }{V}O_{2}$ responses for a representative individual with T2D and a healthy control are presented in Figure 2. Individual $\overset{\cdot }{V}O_{2}$ τp and $\overset{\cdot }{V}O_{2}$ A_s_ responses are provided in Figure 3. The unprimed $\overset{\cdot }{V}O_{2}$ *τ*_p_ and MRT were significantly longer in individuals with T2D compared with healthy controls (*p* < 0.001 for both parameters). PE significantly reduced $\overset{\cdot }{V}O_{2}$ MRT (*p* < 0.001) in the T2D and control groups; however, no group difference was present (*p* = 0.053) during the subsequent exercise transition (diabetes status × condition interaction, *p* < 0.001). PE also elicited a reduction in $\overset{\cdot }{V}O_{2}$ *τ*_p_ in individuals with T2D (*p* < 0.001) but not in the healthy controls (*p* = 0.98) so that $\overset{\cdot }{V}O_{2}$ *τ*_p_ was not different between groups after PE (diabetes status × condition interaction, *p* < 0.001). In addition, subsequent to PE, $\overset{\cdot }{V}O_{2}$ A_s_ was reduced while baseline $\overset{\cdot }{V}O_{2}$ was increased in both the T2D and healthy control groups (condition effect, *p* < 0.001 for both parameters).

**TABLE 2:** Dynamic response characteristics of oxygen uptake ( $\overset{\cdot }{V}O_{2}$ ) at high-intensity cycling exercise transitions.

	Unprimed		Primed	
	Controls	Type 2 diabetes	Controls	Type 2 diabetes
*n*	11	11	11	11
Baseline $\overset{\cdot }{V}O_{2}$ L/min	1.02 ± 0.23	0.90 ± 0.16	1.18 ± 0.23*	0.96 ± 0.20*
$\overset{\cdot }{V}O_{2}$ A_p_, L/min	1.04 ± 0.41	0.79 ± 0.28	1.09 ± 0.38	0.80 ± 0.29
$\overset{\cdot }{V}O_{2}$ *τ*_p_, s	34 ± 2^†^	48 ± 6	34 ± 3	32 ± 6*
$\overset{\cdot }{V}O_{2}$ A_s_, L/min	0.33 ± 0.13	0.26 ± 0.11	0.25 ± 0.14*	0.16 ± 0.07*
$\overset{\cdot }{V}O_{2}$ A_s_, %	24.5 ± 7.0	25.6 ± 10.5	18.5 ± 7.0*	17.6 ± 6.6*
$\overset{\cdot }{V}O_{2}$ TD_s_, s	121 ± 24	138 ± 35	130 ± 26	139 ± 40
$\overset{\cdot }{V}O_{2}$ end A, L/min	2.39 ± 0.61^†^	1.95 ± 0.40	2.52 ± 0.59^†^	1.93 ± 0.45
$\overset{\cdot }{V}O_{2}$ MRT, s	67 ± 5^†^	81 ± 13	57 ± 4*	64 ± 9*
End-exercise $\overset{\cdot }{V}O_{2}$ gain, mL.min^−1^.W^−1^	9.7 ± 1.6	10.1 ± 1.7	9.5 ± 1.2	9.2 ± 1.8

Data are means ± SD; *n* = no. of participants. A, amplitude; τ, time constant; end A, steady-state oxygen uptake ( $\overset{\cdot }{V}O_{2}$ ) response; TD, time delay; *p*, primary phase; s slow component phase. *p < 0.05 vs. unprimed within the same diabetes status group (i.e. within controls or within Type 2 diabetes). ^†^ *p* < 0.05 vs. participants with type 2 diabetes within the same condition (i.e. within unprimed or primed).

### 3.4 Effect of priming exercise on Δ [HHb + Mb] kinetics and tissue oxygenation index responses

**FIGURE 4:**
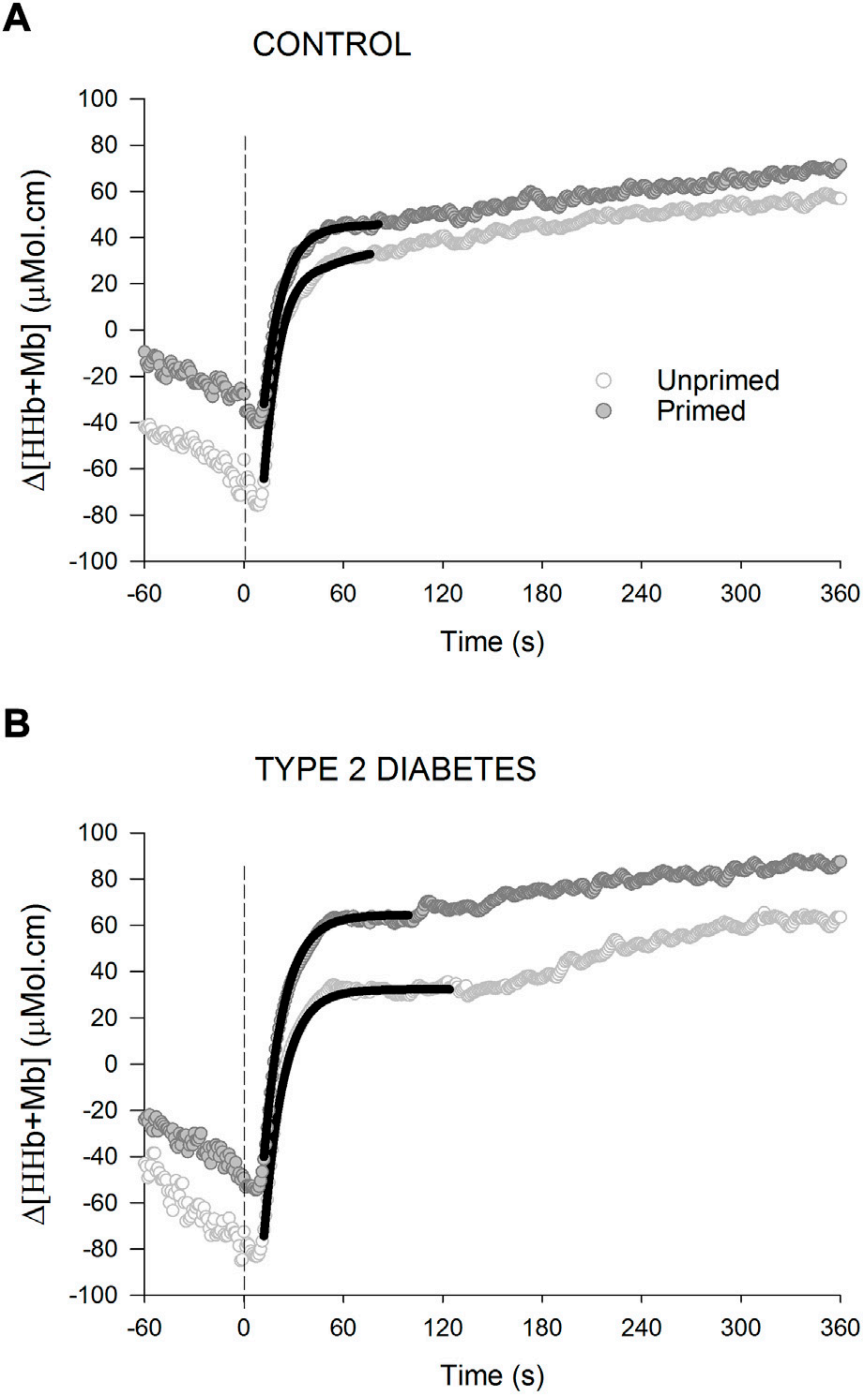
Changes in deoxygenated hemoglobin and myoglobin concentration [Δ (HHb + Mb)] for a representative healthy control **(A)** and an individual with type 2 diabetes **(B)** during high-intensity cycling transitions without priming exercise (open circles) and with priming exercise (solid circles). The vertical line illustrates the abrupt transition to the higher work rate. The continuous black lines of best fit illustrate the primary phase of the Δ (HHb + Mb) response. Note the time constant of the primary phase of the Δ [HHb + Mb] response is not affected by prior priming exercise in any of the 2 groups.

Table 3 presents the parameter estimates for Δ[HHb + Mb] kinetics and TOI responses whereas Figure 4 shows the Δ[HHb + Mb] responses for representative individuals. No statistical group difference was observed in the unprimed parameter estimates. PE resulted in significantly elevated levels of baseline Δ [HHb + Mb] in T2D (*p* < 0.001) and a tendency for higher levels in controls (*p* = 0.08) (group × condition interaction, *p* = 0.04). Participants with T2D showed a larger ratio of the modelled amplitudes of Δ [HHb + Mb]/Δ $\overset{\cdot }{V}O_{2}$ than controls (main effect of group, *p* = 0.01). None of the remaining [HHb + Mb] kinetics parameters were affected by PE. Estimates of baseline TOI as well as ΔTOI were higher during the primed bout in individuals with T2D (*p* < 0.01 for both) but not controls (*p* = 0.7 and 0.9 respectively) (group × condition interaction, *p* = 0.02 and 0.01 respectively).

**TABLE 3:** Dynamic response characteristics of Δ [HHb + Mb] and TOI during high-intensity cycling exercise transitions.

	Unprimed		Primed	
	Controls	Type 2 diabetes	Controls	Type 2 diabetes
*n*	11	11	11	11
Baseline Δ [HHb + Mb], μM*cm	−50 ± 36	−61 ± 62	−33 ± 39	−16 ± 50*
Δ [HHb + Mb] A_p_, μM*cm	90 ± 84	135 ± 75	87 ± 87	141 ± 87
Δ [HHb + Mb] TD_p_, s	10 ± 2	10 ± 2	10 ± 2	11 ± 2
Δ [HHb + Mb] *τ*_p_, s	13 ± 9	12 ± 5	13 ± 7	12 ± 4
Δ [HHb + Mb] τ′, s	23 ± 9	22 ± 5	23 ± 7	23 ± 4
Primary phase Δ [HHb + Mb]/Δ $\overset{\cdot }{V}O_{2}$ A_s_, μM*cm (L/min)	83 ± 63^†^	170 ± 70	83 ± 74^†^	171 ± 95
Δ [HHb + Mb] A_s_, μM*cm	20 ± 17	21 ± 16	21 ± 17	18 ± 16
Baseline TOI, %	75 ± 6	70 ± 5	76 ± 6	74 ± 5*
Δ TOI %	7.4 ± 4.8	6.5 ± 4.5	7.4 ± 5.5	9.3 ± 6.0*

Values are means ± SD; *n* = no. of participants. A, amplitude; τ, time constant; TD, time delay; *p*, primary phase; s slow component phase; τ, time constant; τ′, effective response time (τ + TD); [HHb + Mb], deoxygenated haemoglobin and myoglobin concentration; TOI, tissue oxygenation index; $\overset{\cdot }{V}O_{2}$ oxygen uptake. *p < 0.05 vs. unprimed within the same diabetes status group (i.e. within controls or within Type 2 diabetes). ^†^ *p* < 0.05 vs. participants with type 2 diabetes within the same condition (i.e. within unprimed or primed).

### 3.5 Effect of priming exercise on heart rate kinetics

The HR τ was significantly longer in individuals with T2D compared with healthy controls (main effect of group, *p* < 0.01), but PE did not affect HR τ in any of the groups (T2D unprimed: 56 ± 3 s, T2D primed: 55 ± 2 s; controls unprimed: 51 ± 3 s, controls primed: 50 ± 5 s). Baseline HR was higher in T2D (main effect of group, *p* = 0.04) and subsequent to PE it increased (main effect of condition, *p* < 0.001) in both groups (T2D unprimed: 109 ± 14 beats. min^−1^, T2D primed: 118 ± 13 beats. min^−1^; controls unprimed: 98 ± 9 beats. min^−1^, controls primed: 106 ± 10 beats. min^−1^). End-exercise HR was not different among groups, but it increased following PE (main effect of condition, *p* = 0.017) in both groups (T2D unprimed: 157 ± 12 beats. min^−1^, T2D primed: 159 ± 13 beats. min^−1^; controls unprimed: 156 ± 16 beats. min^−1^, controls primed: 162 ± 15 beats. min^−1^).

## 4 Discussion

In agreement with our primary hypothesis, this study presents for the first-time evidence that in middle-aged individuals with T2D PE reduces $\overset{\cdot }{V}O_{2}$ τp during high-intensity exercise initiated from unloaded exercise without changes in the dynamic response of Δ [HHb + Mb]. In addition, consistent with our second hypothesis, PE significantly reduced the $\overset{\cdot }{V}O_{2}$ A_s_ during the high-intensity exercise bout. Together, these priming exercise-induced effects rendered a reduction in the $\overset{\cdot }{V}O_{2}$ MRT response.

### 4.1 Effect of priming exercise on oxygen uptake τ_p_

In the present study, $\overset{\cdot }{V}O_{2}$ *τ*_p_ responses observed during high-intensity upright cycling transitions were significantly amplified in individuals with T2D (48 s) than controls (32 s) leading to a longer MRT in T2D compared with controls. This is consistent with previous results from Brandenburg et al. (1999) who showed a significantly longer MRT during high-intensity cycling transitions in females with T2D compared with BMI-matched controls (Brandenburg et al., 1999), although Mac Ananey et al. (2011) only observed a tendency for longer $\overset{\cdot }{V}O_{2}$ kinetics in females with T2D of similar characteristics (Mac Ananey et al., 2011). Importantly, we have recently reported in a subgroup of participants who took part in the present study, that T2D slows $\overset{\cdot }{V}O_{2}$ *τ*_p_ during transitions to both, moderate-intensity (Rocha et al., 2019; Rocha et al., 2020) as well as high-intensity work-to-work (Gildea et al., 2021b) transitions. Hence, the present study extends the findings of a blunted $\overset{\cdot }{V}O_{2}$ kinetics response to moderate and high-intensity work-to-work exercise to that of high-intensity exercise initiated from unloaded exercise, at least in middle-aged individuals with T2D when compared with carefully matched healthy controls.

The performance of a PE bout herein resulted in a subsequent significant reduction in this $\overset{\cdot }{V}O_{2}$ *τ*_p_ among individuals with T2D but not in those without, bringing the primed $\overset{\cdot }{V}O_{2}$ *τ*_p_ in T2D in line with the control group. These findings suggest that when the dynamic response of $\overset{\cdot }{V}O_{2}$ is impaired by limitations in O_2_ delivery, as is the case in T2D (Bauer et al., 2007; MacAnaney et al., 2011; Kiely et al., 2014), PE speeds $\overset{\cdot }{V}O_{2}$ *τ*_p_ in the subsequent exercise bout. This notion is supported by studies that have explored these responses when exercising in the prone and supine positions (Rossiter et al., 2001; Jones et al., 2006; Goulding et al., 2017), thus, compromising exercising muscle perfusion pressure and O_2_ delivery (Egaña et al., 2010; Egaña et al., 2013). For instance, an investigation where healthy participants performed high-intensity cycling bouts with and without PE in the supine posture, Jones et al. (2006) observed that PE induced a 37% reduction (*p* < 0.05) in τ $\overset{\cdot }{V}O_{2}$ _p_ (38 s ± 18 s vs. 24 ± 9, s) in the subsequent bout, that was in line with that reported in the unprimed upright posture (Jones et al., 2006). Thus, findings from the current study expand the recently reported findings by our group of a significant speeding in $\overset{\cdot }{V}O_{2}$ *τ*_p_ following priming exercise during moderate-intensity (Rocha et al., 2019; Rocha et al., 2020) as well as high-intensity work-to-work exercise (Gildea et al., 2021b) to that of high-intensity exercise initiated from light exercise in individuals with T2D who are younger than 60 years of age.

The notion that priming exercise enhanced O_2_ supply in the subsequent exercise transition in T2D is evidenced by an increased TOI which suggests an increase in O_2_ availability, likely mediated by a PE-induced increased vasodilation and muscle blood flow at the beginning of the subsequent exercise (Gerbino et al., 1996). However, given that the NIRS-derived overall muscle deoxygenation kinetics and/or amplitude were not affected by PE herein, there is the possibility that the priming-induced reduction in $\overset{\cdot }{V}O_{2}$ *τ*_p_ in T2D was also partly mediated by an improved intracellular O_2_ utilization, likely mediated by the upregulation of rate-limiting mitochondrial oxidative enzymes (Gurd et al., 2006; Gurd et al., 2009) and elevated mitochondrial calcium concentrations (Wüst and Stienen, 2018). On the other hand, the fact that HR kinetics were not altered following PE suggests that central mechanisms (i.e. quicker delivery) did not influence the priming response.

### 4.2 Effect of priming exercise on oxygen uptake slow component

In the present study, PE significantly reduced both $\overset{\cdot }{V}O_{2}$ *τ*_p,_ and A_s_ during the high-intensity transition in participants with T2D. However, in the control group, although PE reduced the $\overset{\cdot }{V}O_{2}$ A_s_ subsequently shortening the overall MRT of the $\overset{\cdot }{V}O_{2}$ response, $\overset{\cdot }{V}O_{2}$ *τ*_p_ remained unaffected. These findings in control individuals are consistent with the current evidence on the influence of PE on subsequent transitions to heavy/severe-intensity upright cycling exercise initiated from an unloaded baseline in healthy participants (Burnley et al., 2000; Burnley et al., 2001; Scheuermann et al., 2001; Burnley et al., 2002a; Burnley et al., 2002b; Fukuba et al., 2002; Burnley et al., 2006; Jones et al., 2006; Goulding et al., 2017)*.*

The priming-induced reduction in the slow component of the present study can likely be attributed to alterations in motor unit recruitment patterns. For instance, our group has recently shown (Gildea et al., 2021b) a priming-induced reduction in iEMG between the end of exercise and the time of the onset of $\overset{\cdot }{V}O_{2}$ A_s_ (ΔiEMG_end_-TD_s_) upon transition to a subsequent high-intensity cycling bout, albeit from an elevated baseline (work-to-work), concomitant with a significant reduction in the $\overset{\cdot }{V}O_{2}$ A_s_. Although, herein, iEMG was not measured, it is possible that PE induced a decreased requirement for additional type II muscle fiber activation during the subsequent high-intensity cycling bout, thereby reducing the associated $\overset{\cdot }{V}O_{2}$ cost of muscle fiber activation (DiMenna et al., 2008). By reducing dependency on these less efficient muscle fibers, the increase in sustained metabolic acidosis, a likely mediator of [PCr] and $\overset{\cdot }{V}O_{2}$ slow components, can be slowed (Poole et al., 1988; Poole et al., 1991; Barstow et al., 1996; Rossiter et al., 2002; Krustrup et al., 2004). Alternatively, PE could facilitate an increased and more homogenous muscle perfusion within the active musculature, which is supported by the observed elevated baseline TOI during the primed bout in T2D herein. Consequently, the reliance on [PCr] degradation and glycogenolysis would be reduced, attenuating the rate of fatigue development and thus, delaying motor unit recruitment (DiMenna et al., 2010). In addition, this altered muscle activation response to priming exercise concomitant with the elevated TOI is consistent with the ‘oxygen-conforming’ effect, which has been demonstrated under involuntary and voluntary small muscle activation (Fitzpatrick et al., 1996; Drouin et al., 2022), although the mechanisms governing the oxygen-conforming response remain to be elucidated.

The observed PE-induced reduction in the $\overset{\cdot }{V}O_{2}$ A_s_ during high-intensity cycling in T2D, is all the more pertinent given individuals with T2D possess a 2-fold increase in type IIb fibers (Mogensen et al., 2007), demonstrate attenuated motor unit firing patterns in the VL compared with healthy controls (Watanabe et al., 2012; Watanabe et al., 2013) and tend to have lower dissociating capacity of myoglobin at intensities above VT (Miyamoto et al., 2020). Nevertheless, it is important to note that not all studies support the association between neuromuscular activation and the $\overset{\cdot }{V}O_{2}$ slow component (Scheuermann et al., 2001; Garland et al., 2006; Cannon et al., 2007), and this is possibly due to the variability associated with measurements and normalization of iEMG.

With this new physiological insight of impaired $\overset{\cdot }{V}O_{2}$ kinetics during high-intensity exercise transitions in T2D that are affected by limitations in O_2_ delivery, future studies should investigate if exercise training mitigates these impairments. This will be practically relevant as high-intensity exercise transitions replicate metabolic transitions akin to those in daily life such as initiating sudden transitions to rapid walking, running, or stair climbing. While recent studies have demonstrated that time-efficient high-intensity interval training as well as longer-duration moderate-intensity continuous exercise training interventions seem effective in enhancing $\overset{\cdot }{V}O_{2}$ *τ*_p_ during moderate-intensity transitions (Green et al., 2020; Gildea et al., 2021a) as well as high-intensity work-to-work transitions (Gildea et al., 2022) in T2D, new studies should explore if these exercise training interventions of different doses can influence the $\overset{\cdot }{V}O_{2}$ kinetics response during high-intensity transitions.

## 5 Limitations

Our results are limited to middle-aged mixed groups of men and women, hence, further studies should explore sex- and/or age-related differences in these outcomes. Even if our protocol did not allow block randomization, the sequence of the unprimed and primed exercise transitions was the same for all participants; hence, this likely has a minor impact on the interpretation of the current findings.

## 6 Conclusion

The present study showed that a preceding high-intensity exercise (i.e. warm-up) or priming exercise accelerated the overall MRT of the $\overset{\cdot }{V}O_{2}$ dynamic response during high-intensity transitions in middle-aged individuals with T2D. This finding was attributed to a speeding of the primary phase time constant of $\overset{\cdot }{V}O_{2}$ and a reduction in the amplitude of the $\overset{\cdot }{V}O_{2}$ slow component while PE did not affect the dynamic response of muscle deoxygenation. Thus, in the presence of the likely diminished vasomotor responses in T2D, it is likely that undertaking a prior high-intensity exercise bout resulted in a more appropriate distribution of blood flow within the working muscle microvasculature, serving to alleviate the metabolic debacle to maintain $\overset{\cdot }{V}O_{2}$ .

## Data Availability

The raw data supporting the conclusions of this article will be made available by the authors, without undue reservation.
